# Multiplexin Promotes Heart but Not Aorta Morphogenesis by Polarized Enhancement of Slit/Robo Activity at the Heart Lumen

**DOI:** 10.1371/journal.pgen.1003597

**Published:** 2013-06-27

**Authors:** Nofar Harpaz, Elly Ordan, Karen Ocorr, Rolf Bodmer, Talila Volk

**Affiliations:** 1 Department of Molecular Genetics, Weizmann Institute of Science, Rehovot, Israel; 2 Development and Aging Program, Sanford-Burnham Medical Research Institute, La Jolla, California, United States of America; University of California San Francisco, United States of America

## Abstract

The *Drosophila* heart tube represents a structure that similarly to vertebrates' primary heart tube exhibits a large lumen; the mechanisms promoting heart tube morphology in both *Drosophila* and vertebrates are poorly understood. We identified Multiplexin (Mp), the *Drosophila* orthologue of mammalian Collagen-XV/XVIII, and the only structural heart-specific protein described so far in *Drosophila*, as necessary and sufficient for shaping the heart tube lumen, but not that of the aorta. Mp is expressed specifically at the stage of heart tube closure, in a polarized fashion, uniquely along the cardioblasts luminal membrane, and its absence results in an extremely small heart tube lumen. Importantly, Mp forms a protein complex with Slit, and interacts genetically with both *slit* and *robo* in the formation of the heart tube. Overexpression of Mp in cardioblasts promotes a large heart lumen in a Slit-dependent manner. Moreover, Mp alters Slit distribution, and promotes the formation of multiple Slit endocytic vesicles, similarly to the effect of overexpression of Robo in these cells. Our data are consistent with Mp-dependent enhancement of Slit/Robo activity and signaling, presumably by affecting Slit protein stabilization, specifically at the lumen side of the heart tube. This activity results with a Slit-dependent, local reduction of F-actin levels at the heart luminal membrane, necessary for forming the large heart tube lumen. Consequently, lack of Mp results in decreased diastolic capacity, leading to reduced heart contractility, as measured in live fly hearts. In summary, these findings show that the polarized localization of Mp controls the direction, timing, and presumably the extent of Slit/Robo activity and signaling at the luminal membrane of the heart cardioblasts. This regulation is essential for the morphogenetic changes that sculpt the heart tube in *Drosophila*, and possibly in forming the vertebrates primary heart tube.

## Author Summary

The formation of the characteristic large heart lumen is common to all heart-containing organisms and is essential for efficient heart function; however, the structural components promoting this process are yet to be elucidated. The *Drosophila* heart represents a specific compartment within an elongated contractile tube, the dorsal vessel, essential for pumping the hemolymph throughout the fly body. Here, we describe a novel extracellular matrix component, Multiplexin (Mp), homologous to vertebrates Collagen XV/XVIII, which is necessary and sufficient for promoting the large heart lumen. Based on molecular and genetic analysis, our findings link Mp activity to a signaling pathway (Slit/Robo) demonstrated previously to repress actin polymerization at the leading edge of migrating neurons. Consistently we show that Mp deposited at the luminal membrane enhances Slit/Robo activity and presumably signaling, leading to reduced actin levels, necessary for curving of the luminal membrane, and for the formation of the large heart lumen. Consequently, *mp* mutant flies exhibit narrow heart and reduced heart contractility. These results demonstrate a novel mechanism by which local deposition of an ECM component promotes a polarized signaling at the luminal aspects of a pair of cardioblasts, shaping the large heart tube compartment.

## Introduction

During early development, the vertebrate heart exhibits genetic and morphological similarities to the cardiac tube (dorsal vessel) of the invertebrate model organism *Drosophila melanogaster* [1]–[3]. The primary genetic network that determines the heart field has been established in both vertebrates and invertebrates [2]. However, the structural components downstream of this primary transcriptional network inducing the large cardiac tube morphology, which differs significantly from that of the aorta, remain to be elucidated.

**Figure 1:**
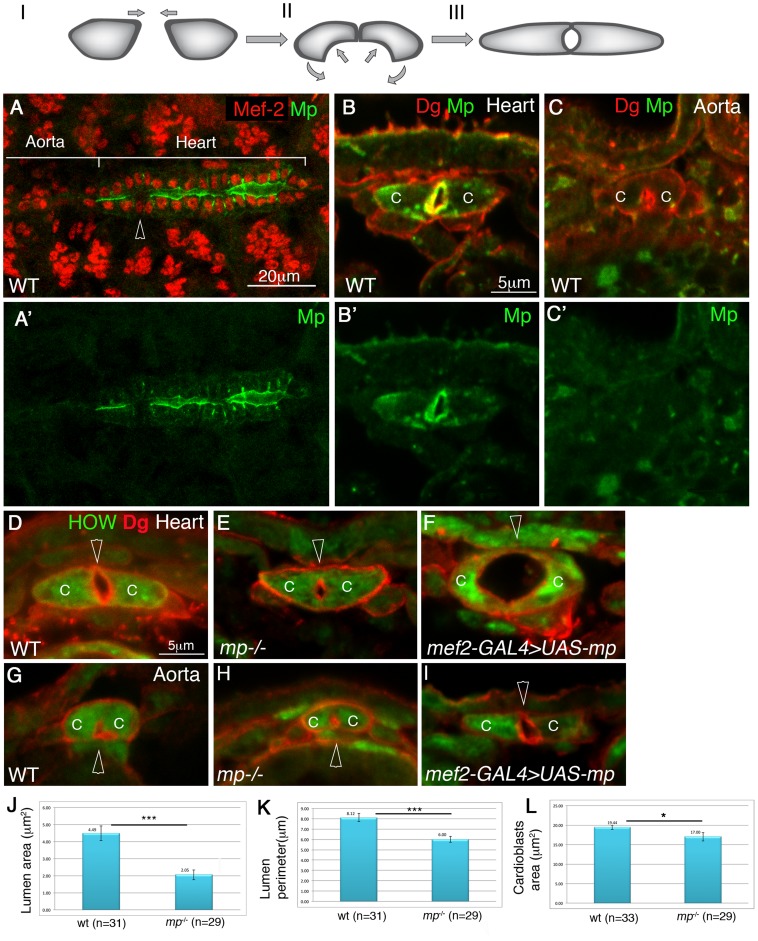
Mp exhibits heart lumen-specific distribution and is necessary and sufficient for cardiac lumen formation. Upper panel: a scheme of the three stages of cardiac tube closure: I- cardioblasts approach the dorsal midline. II- formation of the dorsal junction and the inward curvature of the luminal membrane. III- formation of the ventral junction and tube closure. Mp initial expression is observed between stage II to stage III (Figure S1). A whole mount wild type embryo at stage 16 labeled for Mp (A,A′, green), and Mef-2 (A, red). The heart and aorta domains are indicated. The arrowhead in A indicates a pair of ostia cells which do not express Mp. B-I are cross sections of stage 16 embryos. Wild type heart (B,B′) or aorta (C,C′) labeled with Dystroglycan (Dg, red, B,C) and Mp (green B,B′,C,C′) demonstrate the cardiac specific distribution of Mp in the heart lumen. D-I are cross sections of: wild type heart (D), *mp* mutant heart (E), heart cardioblasts overexpressing Mp (F), wild type aorta (G) *mp* mutant aorta (H), aorta cardioblasts overexpressing Mp (I), labeled with Dg (red) and with anti HOW, which labels the cardioblast cytoplasm (green). Arrowheads in D-I mark the cardiac lumen. Note the formation of large cardiac lumen (29 µm^2^, F), following Mp overexpression in the heart and the formation of a heart-like lumen in the aorta following Mp overexpression in the aorta. J - quantification of the lumen cross section area, K- quantification of the luminal perimeter, L- quantification of cardioblast cross section area measured from 3–4 cross sections per embryo in multiple number of embryos (n). A statistically significant reduction (indicated by three stars) in both cardiac luminal area (reduction of 55%, p = 8.6E-0.6) and perimeter (reduction of 27%, p = 3.8E-0.50) was observed. A slight reduction in cardioblasts total area was also detected (13%, p = 0.02, one star). C- cardioblast cell. Scale bars represent 20 µm in A,A′, and 5 µm in all cross sections (B,C, D–L).

The *Drosophila* dorsal vessel is a single tube, formed by the coalescence of two opposing rows of cardioblasts at the dorsal midline [4]. Following their initial encounter, opposing pairs of cardioblasts contact each other by establishing adherens junctions along the dorsal midline. Subsequently, their future luminal membrane curves inward, creating rows of crescent-shaped cardioblasts. Finally, the ventral-most luminal membrane seals the dorsal vessel tube by forming adherens junctions with opposing cardioblasts and the lumen is formed (Fig. 1, upper panel) [5]. The volume of the lumen of the dorsal vessel depends primarily on two parameters, the length and position of dorsal and ventral adherens junctions formed between pairs of opposing cardioblasts, and the extent of the curvature of the luminal membrane. Importantly, the dorsal vessel is divided into two compartments: the non-contractile anterior aorta, which exhibits an extremely narrow lumen, and the contractile heart domain, characterized by a significantly larger lumen [4]. The genes involved in determining the shape and size of the unique lumen of the heart tube have yet to be characterized.

Previous genetic analyses have identified multiple components contributing to the morphology of the entire dorsal vessel, including both the heart and the aorta compartments. These include Cadherin-mediated adhesion molecules [5], Integrin and its extracellular matrix (ECM) ligand, Laminin [5]–[8], Dystroglycan (Dg) [9], elements of the Slit/Roundabout (Robo) signaling pathway [8]–[11], and the Uncoordinated-5 Netrin receptor [12]. However, all these genes are expressed and required for morphogenesis of the entire dorsal vessel, and thus their activity cannot explain heart-specific morphology. Slit/Robo signaling has been proposed to play a key role in promoting lumen formation by inhibiting cadherin-mediated adhesion [9], [10]. However both proteins are expressed ubiquitously throughout the dorsal vessel, and therefore their activity per se also cannot explain heart-specific morphogenesis. Thus, a structural gene, expressed during the time window of heart tube formation, and localized uniquely at the heart compartment of the dorsal vessel is predicted to mediate heart-specific morphogenesis.

We identified the ECM component, Multiplexin (Mp), a *Drosophila* orthologue of mammalian Collagen XVIII and Collagen XV, as a unique structural protein expressed by the heart but not the aorta cardioblasts of the dorsal vessel. Therefore Mp could be involved in the regulation of heart-specific features. Mp is a secreted multi-domain protein with N-terminal sequences containing Thrombospondin repeats, followed by multiple Collagen repeats, a Collagen trimerization domain, and a C-terminal Endostatin cleavable domain. Mp was described previously in the context of the nervous system and wing development [13], [14]; however, its function in the heart was never been elucidated. Homozygous *mp* mutants are viable but exhibit axonal pathfinding defects detected in the embryonic PNS [13].

Here, we describe a specific role for Mp in *Drosophila* heart tube formation. Our results demonstrate that Mp protein is expressed by the heart, and not the aorta cardioblasts, precisely at the time window of lumen formation. Furthermore, we show that Mp is necessary for heart lumen formation and is sufficient to transform the shape of the aorta tube lumen into that of the heart. Mp executes this function by enhancing Slit/Robo activity presumably by increasing Slit protein stabilization, at the luminal aspects of the heart compartment of the cardiac tube, precisely during the time window of cardiac lumen formation. This leads to the asymmetrical distribution of F-actin along the heart cardioblast membrane essential for the development of the large heart tube lumen. Importantly, the resulting narrow heart tube of *mp* homozygous mutant flies exhibits a physiological defect, as their heart is less efficient in pumping the hemolymph. The results presented here reveal a novel mechanism essential for shaping the large contractile heart tube. Given the remarkable conservation of signaling pathways involved in heart development between flies and vertebrates, and the expression of both collagen XV [15]–[17] and Slit [18], [19] in vertebrates heart it is likely that this mechanism also plays a role in morphogenesis of the vertebrate heart tube.

## Results

### Multiplexin is required for the establishment of the heart-specific lumen

The mRNA of *mp* is expressed in heart cardioblasts at stage 16 at the same time window as cardiac tube formation [13]. In order to analyze Mp protein distribution in the heart, we generated an antibody against the Endostatin domain (see Experimental Procedures). The specificity of the antibody was demonstrated by its lack of reactivity with homozygous *mp* mutants (Supplemental Fig. S1C,C′). Importantly, the antibody staining showed that Mp was specifically localized at the heart lumen, but not at the cardioblast basal domain (Fig. 1A,A′,B,B′). The antibody staining labeled also the border between the hindgut endoderm and the surrounding visceral muscles, glial cells in the CNS, and tendon cells (data not shown). Importantly, the anti Mp antibody did not detect staining at the aorta domain (Fig. 1A,C,C′), although we cannot exclude that the aorta cardioblasts express very low levels of this protein. In addition, Mp was not expressed by the pair of ostia cells (Fig. 1A, arrowhead). Importantly, Mp was only detected in cardioblasts at the stage of tube formation, and not in earlier stages (Supplemental Fig. S1A,B), Thus, Mp exhibits a polarized, heart-specific lumen distribution, during cardiac tube formation.

Analysis of cross sections of the heart from homozygous *mp* mutants revealed a significantly smaller cardiac lumen as compared to controls (Fig. 1D and E). The cross section area and the perimeter of the cardiac lumen of *mp* mutants were about 55% (p = 8.6E-06), and 27% smaller than the wild type (p = 3.8E-05), respectively (Fig. 1J,K). The *mp* mutant cardioblasts appeared slightly smaller than wild type cardioblasts (13% reduction in cardioblast cell area, p = 0.02) (Fig. 1L), but could not explain the significantly reduced lumen size. No difference in lumen size between the aorta of wild type versus that of *mp* mutant was detected (Fig. 1G and H). Thus, the Mp-polarized and specific distribution in the heart lumen, together with the aberrant cardiac morphology in *mp* mutants led us to conclude that Mp function is specific to the future contractile heart compartment of the dorsal vessel, where it is essential for promoting the unique cardiac tube morphology.

### High Multiplexin levels promote a large heart and aorta lumens

To determine whether Mp is sufficient to promote changes in cardiac tube morphology, we overexpressed Mp in the entire dorsal vessel using the muscle-specific driver, *mef2-GAL4*. The distribution of the overexpressed Mp remained polarized at the luminal surfaces (Supplemental Fig. S1D,D′), and it was found to induce a significantly larger cardiac lumen (Fig. 1F), or two ectopic lumens (data not shown). The percentage of embryos showing the large/abnormal lumen was 68% (n = 25). Overexpression of Mp in the aorta led to the enlargement of the aorta lumen to a size comparable to that of the heart (Fig. 1I, Supplementary Fig S1E,F) as detected in 50% of the embryos (n = 8). These phenotypes suggest that high expression of Mp is sufficient to promote enlargement of the luminal membrane domain in both the heart and the aorta compartments.

Taken together, our results suggest that the expression of Mp in the heart luminal domain is sufficient for the induction of the unique shape of the heart cardioblast, and can essentially transform the shapes of the aortal lumen into that typically observed only in the heart compartment.

### Mp interacts genetically with Slit and Robo in promoting heart lumen formation and forms a protein complex with both proteins

**Figure 2:**
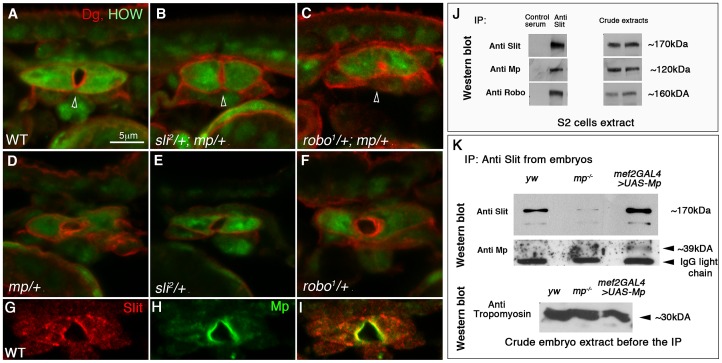
Genetic and physical association between Mp, Slit and Robo. Cardiac cross sections of wild type (A), *slit/+;mp/+* (B), *robo/+;mp/+* (C) double heterozygous, and *mp/+* (D), *slit/+* (E), *robo/+* (F) single heterozygous labeled with anti HOW (green), and anti Dg (red). The cardiac lumens (marked by arrowheads) of the double-heterozygous mutant are smaller relative to the control. G-I: cardiac cross sections of wild type embryos labeled with anti Slit (G,I red) and anti Mp (H,I green), indicating their co-localization along the lumen. J- immunoprecipitation with anti Slit antibodies (or with a control normal mouse serum) of an extract of S2 cells co-transfected with Slit, Robo, and Mp. The same blot was then reacted individually with anti- Slit, Mp, and Robo corresponding to the three upper lanes). The anti Mp antibody reacted with a single band of ∼120 kDa, corresponding to mp cDNA 3hnc1. This immunoprecipitation (IP) is representative of three independent IP experiments. The crude extracts contained comparable amounts of transfected proteins as indicated by the antibody reactivity with each of the transfected cDNA constructs presented in the right panel. K-Immunoprecipitation with anti Slit antibodies of comparable protein extracts taken from stage 16 control (yw), *mp^−/−^*, or embryos overexpressing Mp in heart and muscles (using *mef2-GAL4* driver). Western blot with anti Slit of the IP material shows elevated levels of Slit in the Mp-overexpressing embryos and reduced Slit levels in *mp* mutants. Reaction of the same blot with anti Mp antibodies (lower panel) revealed a specific band of ∼39 kDa, corresponding to the Endostatin fragment. Western blot with anti Tropomyosin of the embryo protein extracts before taken to the IP with Slit, is shown in the lower panel, indicating comparable protein levels in each of the samples.

The narrow cardiac lumen observed in *mp* mutant hearts resembled those that form in *slit* mutants [9]. We therefore tested for genetic interaction between *mp* and *slit* or *robo*. The lumen area of the heart tube of *slit/+;mp/+* double heterozygotes was 48% smaller relative to the wild type heart (p = 0.002, n = 11) (Fig. 2A,B). Moreover, embryos that were double heterozygous for *mp* and *robo* exhibited a lumen area that was 79% smaller than wild type heart (p = 2.9×10^−9^, n = 9) (Fig. 2C). Similarly, the lumen perimeter of *robo^1^/+;mp/+* was 47% smaller than wild type (n = 9), and that of *sli^2^/+;mp/+* was 32% smaller (n = 11) than wild type perimeter. In addition no significant differences were detected between the overall size of the cardioblasts of the double heterozygous relative to wild type ones. Control embryos heterozygous for each mutation alone showed wild type morphology of the cardiac tube (Fig. 2 D,E,F). Based on these results, we concluded that Mp synergizes with Slit and Robo in its function in lumen formation. To determine whether Mp associates with Slit or Robo in a single protein complex, we next tested whether these proteins co-precipitate from extracts of *Drosophila* Schneider S2 cells. We transfected S2 cells with Mp, Slit and Robo cDNAs, and immunoprecipitated Slit with anti Slit antibodies from a soluble extract of the transfected cells. Western blot analysis showed that Slit was specifically immunoprecipitated, and as expected Robo was co-immunoprecipitated as well. In addition, Mp specifically co-precipitated with the Slit/Robo complex (Fig. 2J), suggesting that it forms a protein complex with either Slit, Robo, or with both. Complexes immunoprecipitated with anti Robo antibodies also contained Mp in the co-precipitated material (data not shown). Furthermore, immunoprecipitation of Slit from protein extracts of *mp* mutant embryos, or embryos overexpressing Mp in muscles (using the *mef2-GAL4* driver), showed a significant elevation of endogenous Slit protein levels in embryos overexpressing Mp and a comparable reduction in Slit levels in *mp* mutants, relative to control (Figure 2K). This experiment suggests that Mp might enhance Slit protein stability when both are co-expressed. Interestingly, reaction of the Slit immunoprecipitated material with anti Mp showed a clear band of ∼39 kDa in the lane corresponding to embryos overexpressing Mp, consistent with the association of Slit with the Endostatin domain of Mp. This band was not detected in *mp* mutants, nor in the control yellow/white embryos, possibly because the levels of Mp in the latter embryos are too low for antibody detection. Full length Mp was not detected in any of the IPs with Slit, however the antibody exhibited high background in this region, so we cannot exclude the presence of full length Mp in the IPs. These results are consistent with an effect of Mp on Slit protein stabilization.

Consistent with the co-precipitation experiments, Mp co-localized with Slit at the luminal surfaces of the cardiac tube (Fig. 2G–I). Taken together, these data suggest a functional link between Mp, Slit and Robo in promoting heart lumen formation, and are consistent with the notion that all three proteins are components of a protein complex at the cardioblast luminal membrane.

### The ability of Mp to promote cardiac lumen enlargement is compromised in the absence of Slit

**Figure 3:**
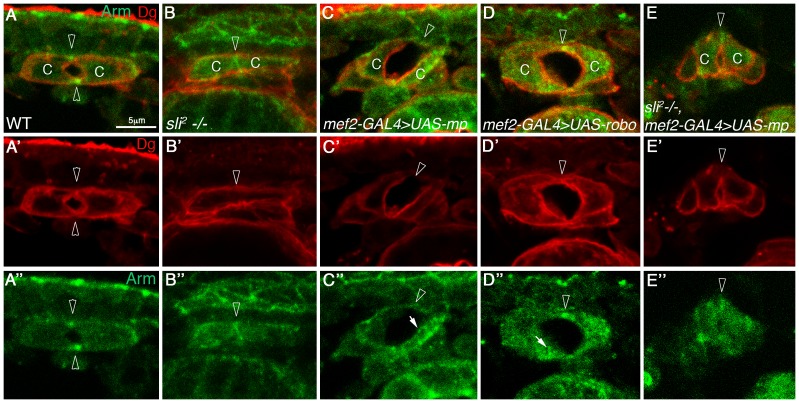
Mp activity in cardioblasts depends on Slit. Cardiac cross sections of wild type (A, A′, A″), *slit* mutants (B, B′, B″), embryos overexpressing Mp in cardioblasts (C, C′,C″), embryos overexpressing Robo in cardioblasts (D, D′,D″), and embryos overexpressing Mp in *slit* mutant background (E, E′, E″), all labeled with anti Dg (red) and with anti Armadillo (green). Arrowheads in A–A″ mark the dorsal and ventral junctions and in B–E″ the dorsal junction. Note that overexpression of Mp is not capable of promoting large and curved lumen in *slit* mutant hearts, and the lack of Armadillo vesicles in these cells. C′ and D′- White arrows mark Armadillo vesicles. C-cardioblast cells. The bar in A is 5 µm and represents the magnification in all panels.

To determine whether Mp activity in the heart requires Slit/Robo signaling, we overexpressed Mp in homozygous *slit* mutants. *slit* mutant cardioblasts lack a well defined lumen (66%), or do exhibit a very small lumen (33%). Staining for beta catenin/Armadillo in *slit* mutant heart often shows a straight line between opposing cardioblasts (Fig. 3,B, B″ arrowhead) and Dg is missing from that region (Fig. 3B′, arrowhead), whereas in the wild type heart, beta catenin/Armadillo is enriched at the dorsal and ventral spots where adherens junctions are formed (Fig. 3 A, A″ arrowheads), and Dg is detected along the entire luminal membrane (Fig. 3A′, arrowheads) [9]. Overexpression of Mp in the wild type embryo led to extension and increased curvature of the luminal membrane (Fig. 3C–C″, arrowheads), an effect that was similar to overexpression of Robo (Fig. 3D–D″). However, overexpression of Mp in *slit* mutant cardioblasts significantly compromised its ability to induce an extended, curved luminal membrane. Either only a very small lumen was detected (in 86% of the mutant embryos (n = 14)) (Fig. 3E–E″, arrowhead), or no lumen was formed between opposing cardioblasts. This suggests that Mp-dependent enlargement of the cardiac tube lumen requires Slit/Robo signaling. Interestingly, although Mp-dependent enlargement of the heart lumen was compromised, Mp was able to partially inhibit accumulation of Armadillo/beta-catenin at the luminal membrane (detected in *slit* mutants), and a concomitant membrane distribution of Dystroglycan reappeared (Fig. 3E′,E″). These results suggest an additional effect of Mp that is Slit independent. We detected multiple cytoplasmic Armadillo vesicles in the cardioblast cytoplasm following overexpression of Mp, or Robo (Fig. 3 C″,D″, white filled arrows), but not in wild type, *slit*, or *slit* overexpressing Mp cardioblasts (Fig. 3 A″,B″ and E″). Taken together these results suggest that Mp activity in promoting a large heart lumen requires Slit/Robo signaling.

### Mp promotes polarized enhancement of Slit/Robo activity at the luminal membrane

**Figure 4:**
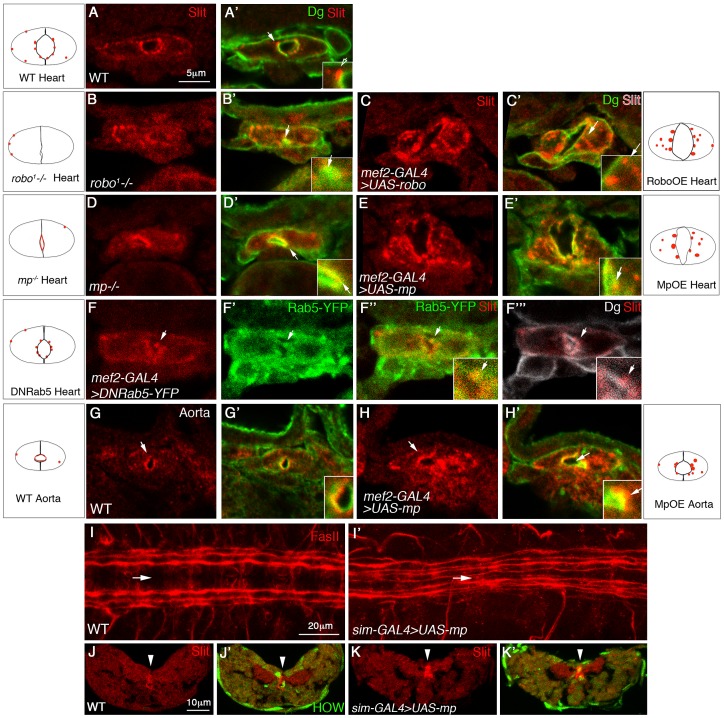
Mp enhances Slit/Robo activity in the heart lumen and modulates Slit distribution in the central nervous system. Cross sections through the heart (A–F′″) or the aorta (G–H′) of wild type embryos (A, A′, G, G′); *robo* mutant (B, B′), embryos overexpressing Robo in cardioblasts (C, C′); *mp* mutant (D, D′), or embryos overexpressing Mp in cardioblasts (E, E′, H, H′) labeled with anti Slit (red) and anti Dg (green). F–F′″ show embryos expressing dominant-negative Rab5-YFP in cardioblasts labeled with Slit (F, red), YFP (F′, green), Slit and YFP (F″), or Slit and Dg (white, F′″). Panels A–H′ are single optical confocal sections to enable comparison of the extent of cytoplasmic Slit vesicles in each genotype. Insets are 2.5 folds enlargement of the heart luminal domain. White arrows in each inset, indicate Slit vesicle/s, with the exception of E′″ where the white arrow indicates Slit vesicle position. Note the reduction in the number and size of Slit endocytic vesicles observed in both *mp* and robo mutants, and their elevation following Mp and Robo overexpression. Represented schemes of Slit vesicles distribution are indicated for each genotype. Note the alteration in Slit distribution following Rab5 dominant negative overexpression (the majority of the protein overlaps Dg at the luminal membrane, and the cytoplasmic Slit is associated with the luminal membrane). I and I′ show wild type (I), or embryo overexpressing Mp in the midline using *sim-GAL4* driver (I′), both labeled for Fasciclin II (red). Note the shortening of the distance between the longitudinal commissures and the midline (marked by the white arrow), following overexpression of Mp. Cross sections through the ventral nerve cord of wild type (J,J′) or an embryo overexpressing Mp in the midline cells (K,K′) labeled with anti Slit (red) and with anti HOW (which marks the midline glia, green J′,K′). High Slit accumulation in the midline (marked by white arrowheads) is observed following overexpression of Mp. Bar in A is 5 µm and represents the magnification in panels A–H′. Bar in I is 20 µm, and represents magnification in I,I′. Bar in J is 10 µm and represents magnification in J–K′).

In wild type heart of a stage 16 embryo, Slit is detected in small cytoplasmic patches, often localized close to the luminal membrane (Fig. 4A,A′). These Slit patches were not described previously in wild type embryos, and could be clearly identified by our new method of visualizing the cardioblasts in cross sections [35]. We found that the number and size of these Slit patches correlated with the extent of Slit/Robo signaling; In *robo^−/−^* cardioblasts the average fluorescent intensity of Slit patches per single cardioblast (calculated as described in M&M), was 36% of that of wild type (p = 0.0015, n = 7) (Figure 4B,B′), whereas in cardioblasts overexpressing Robo, Slit average fluorescent intensity was 2.24 fold higher that that of wild type (p = 0.0005, n = 19) (Figure 4C,C′). Thus, the average fluoresence of cytoplasmic Slit patches represents a measure for the extent of Slit/Robo activity, and can be used to quantify the degree of Slit/Robo signaling.

To verify that the Slit cytoplasmic patches represent endocytic vesicles formed as a result of Slit-Robo interaction at the luminal membrane, we partially arrested endocytosis by cardioblast-specific expression of a dominant-negative version of Rab-5-YFP. This led to detection of slightly larger Slit vesicles, at the luminal membrane, and importantly these vesicles overlapped Rab5-YFP labeling (Fig. 4F–F′″). The lack of these Slit endocytic vesicles in *robo* mutants, and the elevation in their size and number following Robo overexpression further supported the idea that the Slit cytoplasmic “patches” represent endocytic Slit vesicles formed following the binding of Slit to Robo at the luminal membrane. Unfortunately, we could not test for co-localization of Robo and Slit in a given vesicle because both anti Slit and anti Robo antibodies were made in mouse.

Significantly, in *mp* mutant cardioblasts, the average fluorescence of cytoplasmic Slit vesicles was reduced to 61% than that of wild type (p = 0.009, n = 30) (Figure 4D,D′), and Slit distribution was mainly detected at the luminal membrane, overlapping that of Dystroglycan (Dg) (Fig. 4D,D′), supporting the idea that Slit/Robo activity was attenuated. Reciprocally, overexpression of Mp resulted in a 1.8 fold increase of Slit average fluorescence intensity relative to that of wild type (p = 0.0003, n = 27)(Figure 4E,E′). Consistent with the lack of Mp in the aorta, we found fewer cytoplasmic Slit vesicles in this domain (Figure 4G,G′), and Slit was mainly detected at the luminal membrane, overlapping the distribution of Dg similarly to its distribution in *mp* mutant heart cardioblasts (Fig. 4G,G′). However, Mp overexpression in the aorta often led to elevation in the number and size of cytoplasmic Slit vesicles correlating with the enlargement of the aorta lumen (Figure 4,H,H′). Based on the phenotypic similarity between Mp and Robo activities, both in the induction of a large heart lumen, as well as in promoting the formation of Slit cytoplasmic vesicles it was concluded that Mp represents a positive element in the Slit/Robo signaling pathway.

Taken together, these results strongly support our hypothesis that the presence of Mp at the luminal surfaces of the heart tube enhances Slit/Robo activity in a polarized fashion at the luminal domain, and eventually promotes the formation of a large cardiac tube lumen.

### Mp modulates Slit distribution when overexpressed in the CNS

Our results so far demonstrate a tight functional and molecular link between Mp and Slit/Robo signaling in cardiac morphogenesis. To elucidate whether Mp is capable of modulating Slit/Robo signaling in other tissues, and to differentiate between an effect of Mp on the Slit ligand or on its receptor, Robo, we examined the consequences of overexpression of Mp in midline glia cells, which endogenously express Slit [20], but not Robo. Staining of the longitudinal CNS axons with anti Fasciclin II (FasII) in wild type embryos shows three longitudinal axonal tracks on each side of the midline (Fig. 4I). Previous analysis demonstrated that the distance of these tracks from the midline, as well as their position relative to each other, depends on the level of Robo receptors expressed by the axons of each track [21], [22]. Strikingly, overexpression of Mp in midline cells (using the *sim*-GAL4 driver) reduced the distance of the longitudinal axons from the midline, and occasionally led to their defasciculation, forming four tracks (Fig. 4I′). Both phenotypes are consistent with the attenuation of Slit's repulsive activity from the midline, implying a negative effect of Mp on Slit, rather than on Robo. Cross sections of the ventral cord of these embryos, and their labeling with anti Slit antibodies indicated an unusual accumulation of endogenous Slit surrounding the midline glia cells (Fig. 4J–K′, arrowheads), suggesting that ectopic Mp promoted Slit accumulation surrounding the midline glia cells. The midline glia cells are marked with anti Held Out Wing (HOW) shown previously to label these cells [23]. We therefore propose that Mp association with Slit at the surfaces of the midline glia cells inhibits Slit diffusion from the midline glia cells, thus phenocopying a *slit* hypomorphic phenotype. Although the end result of Mp activity in the CNS is opposite to its effect in the heart, in both cases Mp was capable of modulating Slit distribution, presumably by the association of both proteins in a single protein complex, further supporting our hypothesis that by the formation of an Mp-Slit protein complex, Mp elevates local accumulation of Slit, which in the heart, leads to increased binding to Robo.

### Mp activity reduces F-actin levels at the cardiac luminal surfaces in a Slit-dependent manner

**Figure 5:**
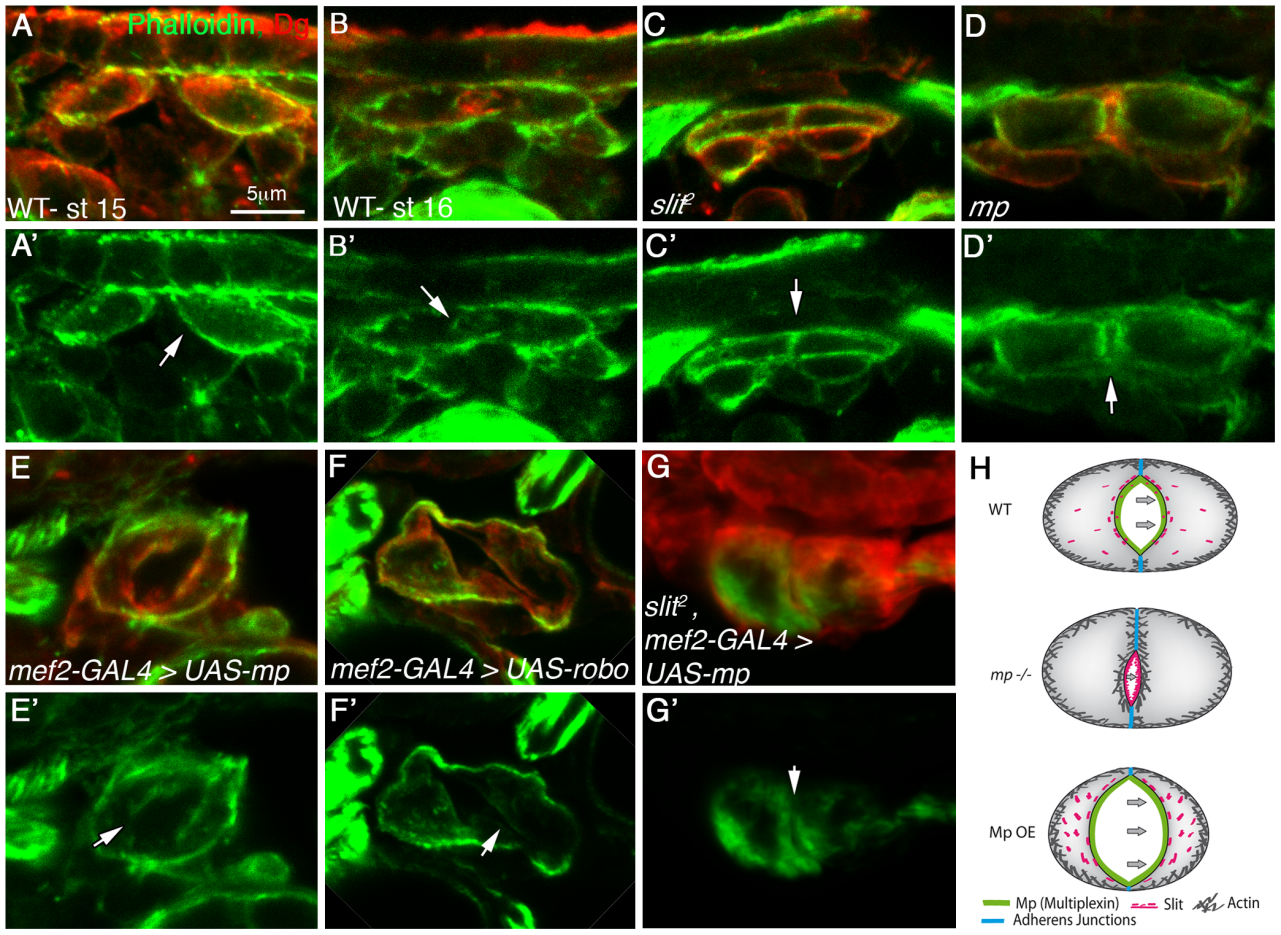
Mp activity reduces F-actin levels at the cardiac luminal membrane in a *slit* dependent manner. Cardiac cross sections of embryos at stage 15 (A, A′) or stage 16 (B–G′) labeled with phalloidin (green) and Dg (red), of the following genotypes: wild type (A, A′, B, B′), *slit* mutant (C, C′), *mp* mutant (D, D′), embryos overexpressing Mp (*mef2-GAL4>UAS-mp*, E, E′), embryos overexpressing Robo (*mef-2-GAL4>UAS-robo*, F, F′) or *slit* mutant embryos overexpressing Mp (G, G′). White arrows indicate the luminal membrane, while the luminal membrane of stage 16 wild type and Mp overexpressing embryos displays reduced F-actin levels, the luminal membrane of *slit* and *mp* mutant exhibits elevated F-actin levels. Note that overexpression of Mp in *slit* mutant background did not reduce F-actin levels at the luminal membrane although a small lumen was detected. The scheme in H summarizes the results; in wild type (WT) heart a constitutive activation of Slit/Robo at the luminal membrane, promoted by Mp reduces F-actin levels at the luminal membrane. In *mp* mutants Slit/Robo signaling is reduced, and consequently F-actin levels are elevated leading to a small lumen. In contrast, overexpression of Mp (Mp OE) leads to elevated Slit/Robo signaling, reducing luminal F-actin levels and enhancing lumen size. Mp overexpression in the absence of Slit exhibited elevated levels of luminal F-actin and a small lumen. Bar in A is 5 µm and represents magnification of all panels.

In axons, Slit/Robo signaling counteracts actin polymerization, leading to membrane retraction [24]–[26]. Therefore, we investigated the relative levels of F-actin in the luminal versus basolateral surfaces of heart cardioblasts. At stage 15, the entire cardioblasts surface showed positive phalloidin staining (Fig. 5A,A′). At stage 16, when the cardiac lumen has been established, we detected a significant reduction of F-actin at the luminal surfaces, while the basolateral levels of F-actin remained high (Fig. 5B,B′). Significantly, in both *slit*, and *mp* mutant embryos, there was no difference between the luminal and basal F-actin levels as observed in wild type (Fig. 5C,C′,D,D′). This difference could not be attributed to the close proximity of opposing luminal membranes because it was observed even when a partial small lumen had been formed in *mp* mutant embryos (Fig. 5D′). Furthermore, overexpression of Mp in cardioblasts led to extension of the luminal membrane as well as a significant reduction of F-actin at the luminal domain (Fig. 5E,E′ arrow), similar to the effect obtained following overexpression of Robo (Fig. 5F,F′ arrow). In both cases, reduced F-actin correlated with the formation of an extended luminal membrane. Importantly, overexpression of Mp in *slit* mutant cardioblasts did not lead to reduction of luminal F-actin relative to its basolateral levels (as in wild type hearts), suggesting that this reduction is dependent on Slit (Fig. 5G,G′). These results demonstrate that the Slit-dependent Mp activity in promoting enlargement of the heart lumen compartment correlates with a significant inhibition of F-actin polymerization along the luminal membrane.

### The hearts of homozygous mp mutant flies exhibit reduced contractile activity

**Figure 6:**
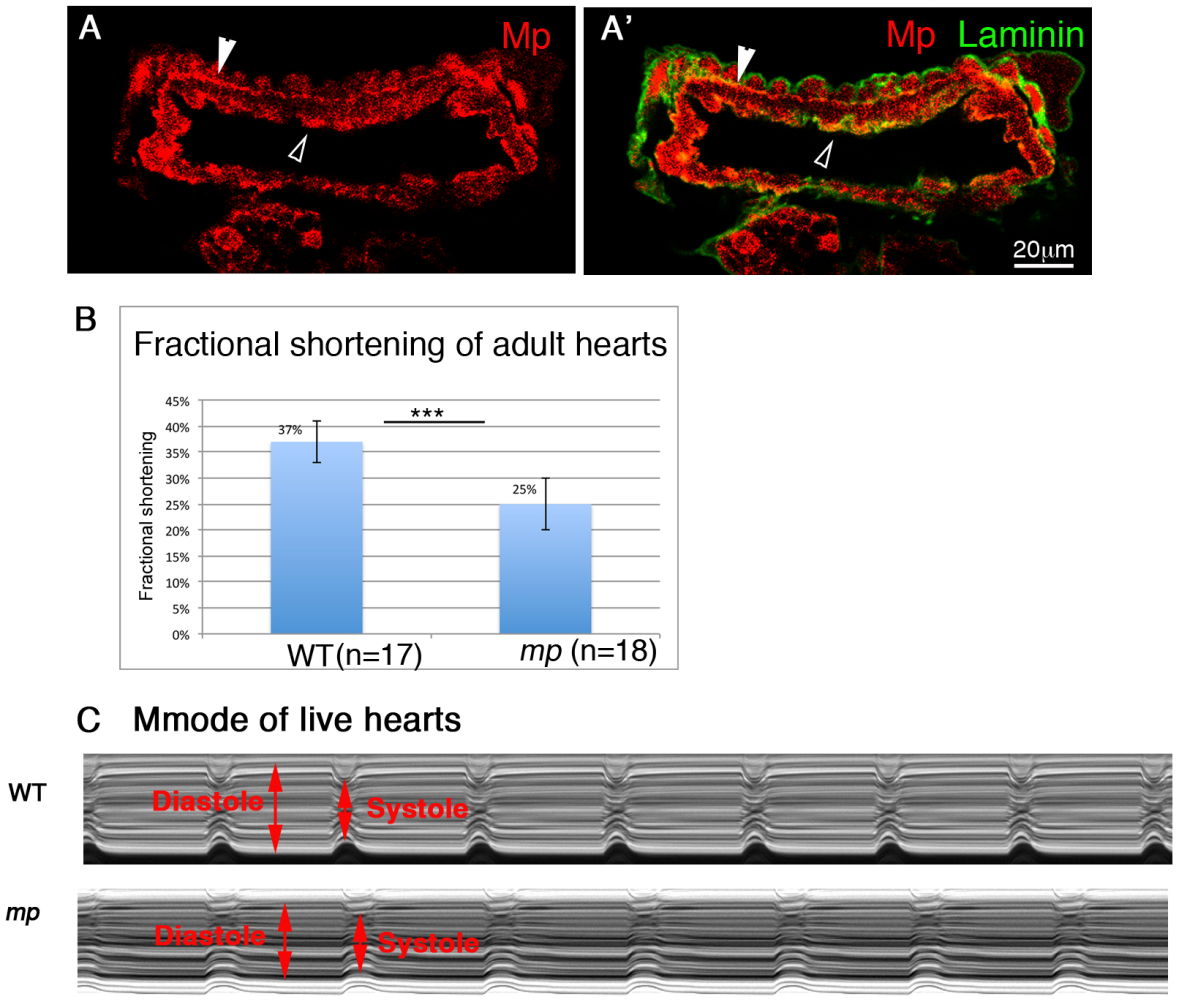
Mp activity is essential for proper heart contraction of adult fly hearts. Cardiac cross sections through an adult heart stained with anti Mp (red, A,A′) and anti Laminin (green, A′). Empty white arrowheads in A and A′ indicate positive staining for Mp in the cardiac lumen. Filled white arrowheads show positive staining of Mp between the cardioblast layer and the ventral longitudinal muscles. B- Heart contractility is quantified as fractional shortening or the extent to which the heart walls come together during systole. Fractional shortening is significantly reduced in *mp* mutant flies. C- the reduction in fractional shortening is also evident in representative Mmode records from wild type (WT) or *mp* mutant hearts. Diastole and systole levels are indicated by red arrows.

Finally, we determined whether the morphological defects observed in *mp* mutant embryos are of physiological significance for the functional contracting heart of the adult fly. The heart in flies does not undergo extensive histolysis, and the embryonic cardioblasts maintain their identity throughout development. Mp distribution was maintained in the adult fly heart, where it could be detected both along the luminal membrane, as in the embryo (Fig. 6A,A′, white empty arrowheads), as well as at the contact area between the ventral muscles and the cardiac tube (Fig. 6A,A′, white filled arrowhead). In both cases it overlapped the Laminin distribution. The physiological relevance of the aberrant heart morphology detected in the embryonic heart of *mp* mutants was analyzed by measurements of live contracting hearts of *mp* mutants as well as of wild type adult hearts [27] (see also representative videos in Supplementary Material, movie S1). We were specifically interested in the effects of *mp* on heart contractility, given the reduction in lumen size observed in *mp* mutants. This can be quantified as the percent fractional shortening, which is essentially the extent of contraction during systole compared to diastole ([diastolic diameter-systolic diameter]/diastolic diameter). We observed (Fig. 6B) a significant reduction (25% versus 37% P<0.005) in the percent fractional shortening in *mp* mutants, indicating reduced contractility of the heart tube. This is also evident in M-mode records from these hearts, which show the movement of the heart walls (y-axis) over time (x-axis). These movements are much shallower in *mp* mutant hearts, resulting from a decreased ability of the heart walls to retain full diastolic values (Fig. 6C). A video of representative heart contraction of *mp* mutant fly is shown in Supplementary information (movie S2).

Therefore, these results indicate that Mp is essential for proper heart contraction, and in its absence, the pumping efficiency of the hemolymph in the adult fly is compromised.

## Discussion

The structural genes mediating heart tubular morphogenesis have not yet been elucidated. Here, we describe a gene, *mp*, expressed in the heart at the time window of cardiac tube formation, which is localized at the heart but not the aorta tube surfaces, and mediates the formation of the characteristic heart lumen. Furthermore, our analysis uncovered a novel molecular network involving Mp, Slit, Robo, F-actin, and beta-catenin that specifically promotes heart tube morphology.

**Figure 7:**
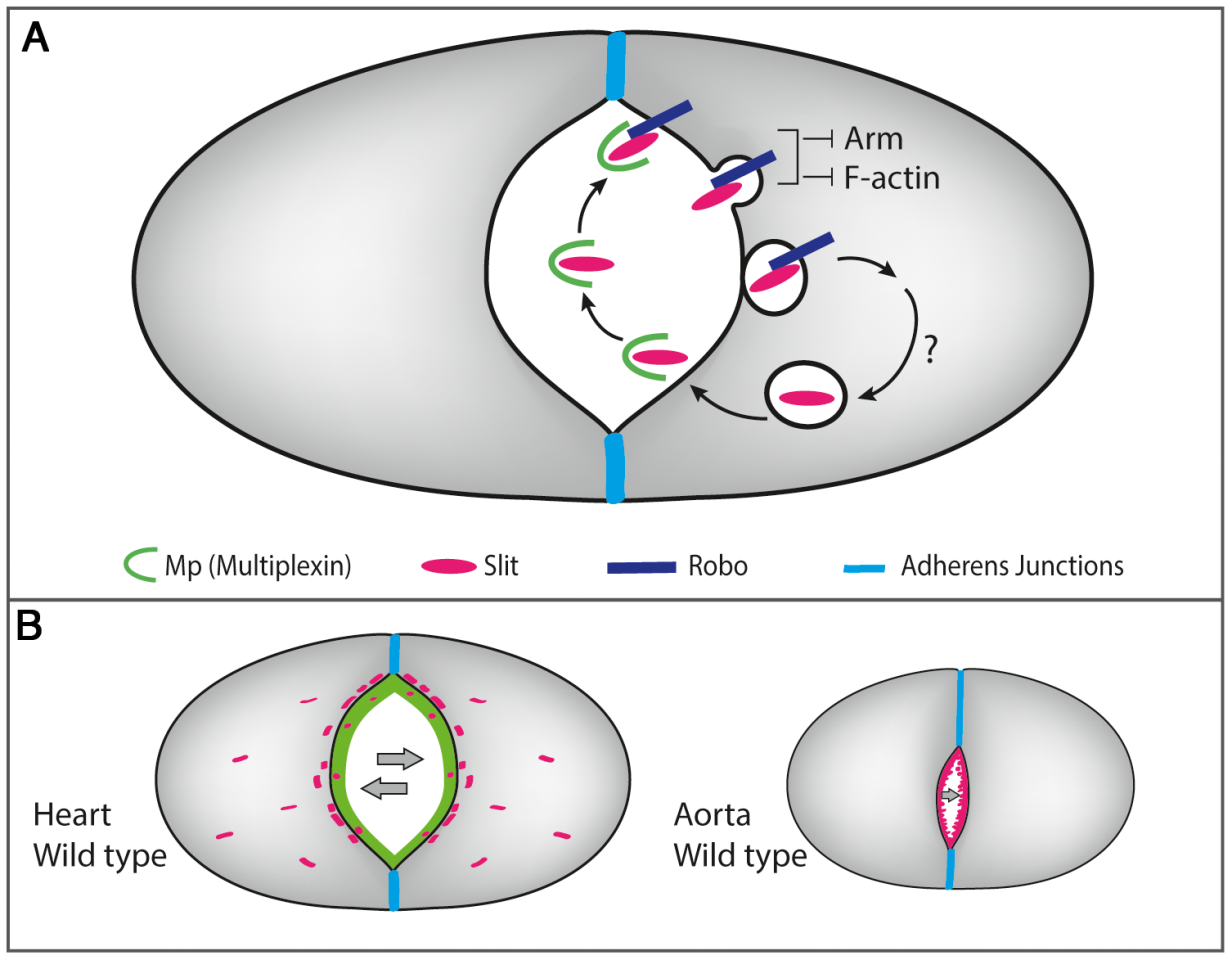
A scheme describing the molecular interactions between Mp, Slit/Robo and F-actin in the heart and the aorta. A- Heart-specific expression of Mp that is restricted to the luminal membrane, promotes the formation of Mp-Slit complex facilitating Slit/Robo activity, leading to the formation of Slit endocytic vesicles close to the luminal membrane. Slit/Robo signaling reduces F-actin levels at the luminal membrane, and restricts the junctional domain by promoting Armadilo turn over. Reiterated Slit/Robo signaling is maintained as a result of continued Mp expression and its association with newly secreted Slit, leading to inward curving of the luminal membrane. B- Lack of Mp in the aorta limits Slit/Robo activation, leading to a significantly narrow lumen.

We suggest that polarized deposition of Mp at the luminal extracellular space of the heart, promotes Mp-dependent accumulation of Slit (as was shown in the CNS), leading to the enhancement of Slit/Robo activity (measured by elevated Slit endocytosis), which consequently promotes enhanced polarized Slit/Robo signaling at the heart luminal membrane. As a result of this asymmetric Slit/Robo signaling, the homogenous distribution of F-actin along the entire cardioblast membrane, observed prior to tube closure, is shifted towards an asymmetric distribution in which reduced F-actin is induced at the luminal membrane, mediating inward membrane curving and the formation of a larger lumen (see model in Fig. 7 A). The cross section area of the cardiac lumen is essentially determined by two factors, the distance between the dorsal and ventral junction domains, and the extent of luminal curving. Our data suggest that Mp is involved in both these processes. Thus, spatially and temporally controlled Mp expression in the heart compartment shifts Slit/Robo signaling despite the uniform distribution of both Slit and Robo in the entire dorsal vessel, restricting the signaling event to the luminal membrane of the heart compartment (see model in Fig. 7 B).

### Mp enhances Slit/Robo activity by affecting Slit distribution and stability

Because the aortal domain develops a lumen (although small) in the absence of Mp, we favor the idea that while Slit/Robo signaling is critical for lumen formation per se, Mp activity modulates this signaling in a polarized fashion, eventually leading to higher level of activation of Slit/Robo signaling at the heart luminal membrane. The precise time window of Mp expression, which correlates with the timing of cardiac lumen formation and its polarized luminal distribution are both critical for cardiac-specific tube morphogenesis, as both Slit and Robo distributions are wider and extend along the entire dorsal vessel. The significant reduction in Slit endocytic vesicles in *mp* mutants and their enhanced appearance following Mp overexpression implicate Mp as a critical factor in promoting the constitutive local activation of Slit/Robo signaling.

Unlike the situation in the nervous system, Slit in the cardioblasts acts in an autocrine, cell autonomous manner [9], [10], and its activity appears to be continuous (as judged by its multiple endocytic vesicles). We therefore suggest that Mp, expressed in a tightly controlled manner in the heart, is required for regulating Slit activity. Excessive signaling leads to an aberrant, often opened tube as observed in Mp overexpression, and was also reported following Robo overexpression [10]. On the other hand, reduced Slit signaling leads to a very narrow tube as seen in distinct *robo, mp* or *slit* mutants. The role of Mp as a heart-specific local enhancer of the Slit/Robo signaling pathway is supported by the following findings: (a) It co-localizes with Slit on the heart luminal surfaces. (b) It forms a protein complex with Slit in S2 cells and in embryos. (c) It appears to enhance Slit protein stability. (d) In its absence, Slit endocytic vesicles are significantly reduced (as in the *robo* mutant). (e) Mp overexpression leads to supernumerary Slit endocytic vesicles (similar to the Robo overexpression phenotype). (f) Its ability to induce an extended cardiac tube lumen is suppressed in *slit* mutants. (g) It facilitates Slit accumulation in the midline following its overexpression.

The effect of Mp on Slit might be explained on a molecular level by local enhancement of Slit protein stability, demonstrated by elevation of endogenous Slit levels, and/or accumulation following Mp overexpression. Slit binding to chondroitin-sulfate chains of Syndecan has been shown to increase its affinity for Robo [28]. Mp proteins contain chondroitin sulfate chains [14], and thus could bind directly to Slit and Robo via these chains. This can promote local accumulation of Slit at the luminal membrane and enhance Slit/Robo interactions. Alternatively, Mp might facilitate the interaction between Slit and Syndecan, which was previously shown to contribute to heart morphology [29]. The latter possibility is less likely because we did not find genetic interaction between *syndecan* and *mp* in double heterozygous embryos and the hearts of the latter was normal (N. Harpaz, data not shown). Our results suggest that whereas Slit is epistatic to Mp, overexpression of Robo in the aorta is capable of promoting expansion of the aorta, even in the absence of Mp (data not shown), implicating that Mp is not absolutely required for Slit/Robo activation.

### Mp cooperates with Slit in promoting reduced F-actin levels at the luminal membrane

Previous studies suggested that the Slit/Robo signaling pathway counteracts the formation of adherens junctions at the luminal surfaces [9], [10]. However, the ubiquitous distribution of Slit along both the aorta and heart domain, and its earlier function in promoting the polarity of the cardioblasts prior to the formation of the tube [11], cannot explain the direct involvement of Slit in the specific formation of the heart lumen. Mp, on the other hand, is secreted to the luminal ECM during tube closure and lumen formation, and therefore most likely plays a specific role during this stage.

Our results are consistent with a major function for Slit/Robo signaling in reducing F-actin levels at the luminal surfaces, thus leading to asymmetrical distribution of F-actin along the cardioblast membrane. Asymmetric distribution of F-actin as a result of cell confinement or differential adhesion has been recently shown to promote spontaneous lumen formation in MDCK cells [30], supporting our observations. In addition, reduced F-actin at the luminal membrane might have a dual function for cardiac tube lumen formation: It could restrict the domain of dorsal and ventral adherens junctions, allocating more luminal membrane at the expense of junctional membrane, and it may facilitate the inward curvature of the luminal membrane. This latter effect is probably tightly controlled, as extended curvature leads to the formation of lumen inside a single cardioblast as observed in cells overexpressing Mp.

The inhibitory effect of Mp on beta-catenin was deduced from the accumulation of Armadilo vesicles following Mp overexpression, as was similarly observed following Robo overexpression. In vitro studies previously linked Slit/Robo signaling to reduced N-cadherin adhesion resulting in detachment of beta-catenin from N-cadherin [31]. A direct, Slit-independent effect of Mp on beta-catenin is possible, as a recent study showed that Mp promotes a decrease of the extracellular Wg/Wnt protein in the *Drosophila* proventriculum zone [14] and the C-terminal cleaved polypeptide Endostatin has consistently been shown to inhibit Wnt signaling, by promoting beta catenin degradation in vertebrates [32].

### A link to CollagenXV/XVIII-related human diseases

Both mammalian Mp orthologue genes, Collagen XV and Collagen XVIII, have been linked to various diseases. Collagen XV had been recently associated with predisposition to cardiomyopathy [16], [17] and similarly the Slit/Robo signaling pathway has been linked to cardiac morphogenetic defects [19], it is possible that these two proteins functionally interact to promote cardiac morphogenesis. A Collagen XVIII deficiency leads to Knobloch syndrome, which is characterized by various pathologies, including viteoretinal degeneration with retinal detachment as well as a neural tube closure defect [33], [34]. Whereas no heart defects were described in the *col18a* mutant mice, or in humans with Knobloch syndrome, a possible functional connection between ColXVIII and the Slit/Robo signaling pathway might exist. Finally, similarly to Collagen IV shown to modulate TGF-β signaling, [35], [36], Mp appears to modulate Slit signaling by controlling its levels.

In summary, our analysis revealed a novel mechanism of cardiac tube lumen morphogenesis associated with the polarized tissue-specific activity of *mp*, the *Drosophila* orthologue of the *col18a and col15* genes. Our study explains how Mp interaction with the Slit/Robo signaling pathway promotes the inward curvature of the luminal membrane specifically in the heart domain.

## Materials and Methods

### Fly strains

Wild type flies were *yw* or *hand-GMA-GFP* (obtained from F. Schnorrer, MPI, Martinsried, Germany), *mp* mutants were *dmp^ΔN4-11^*, and *UAS-Mp-3hnc1* (obtained from B. Moussian, MPI, Tubingen, Germany) and were described in [13], *sli^2/^CyO,Yfp* and *robo^1/^CyO, Yfp* obtained from B.J. Dickson, Research Institute of Molecular Pathology, Austria, and *UAS-Robo* was obtained from SG. Kramer, UMDNJ-Robert Wood Johnson Medical School described in [10]. GAL4 lines were *mef2-GAL4* and *sim-Gal4* (Bloomington stock center, Indiana, USA). For the induction of Rab5-dominant negative, a UAS-Rab5-DN-YFP line was obtained from the Bloomington stock center, Indiana, USA. For the induction of Mp over expression, two copies of the *mef2-GAL4* and two copies of the *UAS-Mp* were used (at 25°C), with the exception of Fig. 1K, where one copy of each construct was used but the embryos were raised at 29°C. For Robo over expression, single copies of *mef2-GAL4* and *UAS-Robo* were used. Selection of the *sli^2^* and *robo^1^* homozygous embryos was done on the basis of a YFP-containing balancer. The selection of *sli^2^* mutant embryos overexpressing Mp, was based on an aberrant CNS phenotype.

### Antibodies and reagents used for immunoflorescence staining

Primary antibodies used included: rabbit anti Mef2 (1∶200, Nguyen H.T, University of Erlangen-Nuremberg, Germany), rat anti Mp (1∶100 for whole embryos, 1∶35 in sections) was produced in our lab by rat immunization of a purified GST fused to the Endostatin domain of Mp, mouse anti Fasciclin II (1∶10, this monoclonal antibody was developed by Goodman, C., was obtained from the Developmental Studies Hybridoma Bank, developed under the auspices of the NICHD and maintained by The University of Iowa, Dept of Biology, Iowa City, IA 52242); rabbit anti Dg (1∶140, a gift from W.M. Deng, Florida State University, FL, USA) [37], rat and rabbit anti HOW (1∶35, were produced in our lab), guinea pig anti Laminin (1∶70, produced in our lab), mouse anti Slit (1∶10, this monoclonal antibody was developed by Artavanis-Tsakonas, S., was obtained from the Developmental Studies Hybridoma Bank, developed under the auspices of the NICHD and maintained by The University of Iowa, Dept of Biology, Iowa City, IA 52242DSHB), mouse anti Armadillo (1∶70, this monoclonal antibody was developed by Wiescjaus, E. was obtained from the Developmental Studies Hybridoma Bank, developed under the auspices of the NICHD and maintained by The University of Iowa, Dept of Biology, Iowa City, IA 52242DSHB), chick anti GFP (1∶70, Aves Labs, Inc). Alexa Fluor 488 phalloidin was used (1∶140, Invitrogen, A12379) for F-actin labeling. Secondary antibodies conjugated with Cy2, Cy3, Cy5 or 405 raised against guinea pig, rat, rabbit, mouse and chick were purchased from Jackson Laboratories, Inc.

### Embryos preparation for cross section and whole mount immune-staining

Staged embryos were collected, dechorionated and fixed as described previously [38]. For phalloidin staining EtOH instead of MeOH was used during fixation, otherwise embryos were fixed as described in Gilsohn and Volk [39].

Confocal images were taken with a Zeiss LSM710 confocal system, and processed with Adobe Photoshop.

### Quantification of Slit vesicles fluorescence

The Fiji/ImageJ program was used to quantify the fluorescence intensity of cytoplasmic Slit in cardioblasts. A threshold for vesicles fluorescence was defined (according to the signal/background ratio) on average intensity projection lsm images, and the amount of fluorescence within each of the cardioblasts was calculated as the percentage of the vesicles area relative to the total area of the cardioblast (visualized by Dg staining). The fluorescence intensity was normalized to that of wild type cardioblast. The statistical significance of the measurments was calculated by student T test.

### Measurements of the adult heart

The analysis was performed as described previously [27], [40]. In brief, flies (1–2 week old) were dissected and their beating hearts were filmed using high speed digital cameras (EM-CCD digital camera, Hamamatsu Corp) and light microscopy. Heart measurements and calculation of physiological parameters were performed with the HC image data capture software (Hamamatsu Corp).

The percentage of fractional shortening was calculated as the difference between the diastolic and the systolic ranges, divided by the diastolic range. The result was multiplied by 100.

### Measurements of lumen dimensions in embryos cross sections

Lumen and cardioblasts dimensions were measured using the Zen 2008 Confocal program. The measurements were taken from lsm projection images, using the open/closed Bezier tool.

### Tissue culture and immunoprecipitation

Constructs used for cells transfection included: *pUAST-robo-HA* (BJ Dickson, Research Institute of Molecular Pathology, Austria), *pUAST-slit* (GJ. Bashaw, University of Pennsylvania School of Medicine, Philadelphia, USA) and *pUAST-mp* (B. Moussian, Max-Planck Institute, Germany).

SR+ cells were transfected all 3 cDNA constructs (Robo, Slit, Mp) using the Escort IV transfection reagent (Sigma-Aldrich, # L3287), and grown for 36 hours. The cells were pelleted and lysed in NP40 0.5% containing PBS. The soluble lysate was incubated with protein A/G beads (SC-2003, Santa Cruz) pre-coupled with mouse anti Slit antibody or with normal mouse serum as a control, over night at 4°C, washed 3 times with the extraction buffer, and boiled in sample buffer, and separated by SDS polyacrylamide gel electrophoresis. For embryos protein extract analysis, stage 16 embryos were collected and lysed with RIPA buffer. For Western blot analysis, proteins were transferred onto a nitrocellulose membrane, blocked with 5% milk in PTW, reacted with the primary antibody (2 hrs, at RT), washed in PBSX3 and then reacted with the secondary antibody conjugated to HRP. ECL reaction was performed after multiple washings of the nitrocellulose membrane.

## Supporting Information

Figure S1 Mp distribution during heart tube formation, and its ability to expand the lumen of the aorta. Cross sections of cardioblasts at early stage 15 prior to cardioblasts encounter (A,A′) and following the formation of the dorsal junction (B,B′) stained for HOW (blue, marks the cardioblast cytoplasm), Mp (green) and Laminin (red). The arrowheads in B and B′ indicate the initial luminal secretion of Mp which partially overlaps Laminin staining. C,D Cross section of *mp* mutant heart (C,C′), or cardioblasts overexpressing Mp (D,D′), with anti Mp antibodies (green C–D′) and with anti Dg (red C,D). The specificity of the antibody is demonstrated by its negative staining in *mp* mutant. Note that overexpression of Mp in cardioblasts does not change its polarized luminal distribution (D). E,F Dorsal view of whole embryos labeled with anti Mef2 (red) and anti Laminin (lan, green). E-wild type embryo, F-embryo overexpressing Mp. Arrows indicate the aorta and arrowheads indicate the heart domains. Note the expansion of the aorta lumen following overexpression of Mp.
(TIF)

Movie S1 The movie represents 30 seconds high-speed video of contracting heart in live adult 1–2 weeks old wild type female.
(MP4)

Movie S2 The movie represents 30 seconds high-speed video of contracting heart in live adult female fly 1–2 weeks old of *mp* homozygous mutant fly.
(MP4)

## References

[1] Zaffran S, Frasch M (2002) Early signals in cardiac development. Circ Res 91: 457–469. 12242263 10.1161/01.res.0000034152.74523.a8

[2] Olson EN (2006) Gene regulatory networks in the evolution and development of the heart. Science 313: 1922–1927. 17008524 10.1126/science.1132292 PMC4459601

[3] Bier E, Bodmer R (2004) Drosophila, an emerging model for cardiac disease. Gene 342: 1–11. 15527959 10.1016/j.gene.2004.07.018

[4] Tao Y, Schulz RA (2007) Heart development in Drosophila. Semin Cell Dev Biol 18: 3–15. 17208472 10.1016/j.semcdb.2006.12.001

[5] Haag TA, Haag NP, Lekven AC, Hartenstein V (1999) The role of cell adhesion molecules in Drosophila heart morphogenesis: faint sausage, shotgun/DE-cadherin, and laminin A are required for discrete stages in heart development. Dev Biol 208: 56–69. 10075841 10.1006/dbio.1998.9188

[6] Yarnitzky T, Volk T (1995) Laminin is required for heart, somatic muscles, and gut development in the Drosophila embryo. Dev Biol 169: 609–618. 7781902 10.1006/dbio.1995.1173

[7] Vanderploeg J, Vazquez Paz LL, MacMullin A, Jacobs JR (2012) Integrins are required for cardioblast polarisation in Drosophila. BMC Dev Biol 12: 8. 22353787 10.1186/1471-213X-12-8 PMC3305622

[8] MacMullin A, Jacobs JR (2006) Slit coordinates cardiac morphogenesis in Drosophila. Dev Biol 293: 154–164. 16516189 10.1016/j.ydbio.2006.01.027

[9] Medioni C, Astier M, Zmojdzian M, Jagla K, Semeriva M (2008) Genetic control of cell morphogenesis during Drosophila melanogaster cardiac tube formation. J Cell Biol 182: 249–261. 18663140 10.1083/jcb.200801100 PMC2483531

[10] Santiago-Martinez E, Soplop NH, Patel R, Kramer SG (2008) Repulsion by Slit and Roundabout prevents Shotgun/E-cadherin-mediated cell adhesion during Drosophila heart tube lumen formation. J Cell Biol 182: 241–248. 18663139 10.1083/jcb.200804120 PMC2483515

[11] Qian L, Liu J, Bodmer R (2005) Slit and Robo control cardiac cell polarity and morphogenesis. Curr Biol 15: 2271–2278. 16360689 10.1016/j.cub.2005.10.037

[12] Albrecht S, Altenhein B, Paululat A (2011) The transmembrane receptor Uncoordinated5 (Unc5) is essential for heart lumen formation in Drosophila melanogaster. Dev Biol 350: 89–100. 21094637 10.1016/j.ydbio.2010.11.016

[13] Meyer F, Moussian B (2009) Drosophila multiplexin (Dmp) modulates motor axon pathfinding accuracy. Dev Growth Differ 51: 483–498. 19469789 10.1111/j.1440-169X.2009.01111.x

[14] Momota R, Naito I, Ninomiya Y, Ohtsuka A (2011) Drosophila type XV/XVIII collagen, Mp, is involved in Wingless distribution. Matrix Biol 30: 258–266. 21477650 10.1016/j.matbio.2011.03.008

[15] Muona A, Eklund L, Vaisanen T, Pihlajaniemi T (2002) Developmentally regulated expression of type XV collagen correlates with abnormalities in Col15a1(−/−) mice. Matrix Biol 21: 89–102. 11827796 10.1016/s0945-053x(01)00187-1

[16] Rasi K, Piuhola J, Czabanka M, Sormunen R, Ilves M, et al (2010) Collagen XV is necessary for modeling of the extracellular matrix and its deficiency predisposes to cardiomyopathy. Circ Res 107: 1241–1252. 20847313 10.1161/CIRCRESAHA.110.222133

[17] Eklund L, Piuhola J, Komulainen J, Sormunen R, Ongvarrasopone C, et al (2001) Lack of type XV collagen causes a skeletal myopathy and cardiovascular defects in mice. Proc Natl Acad Sci U S A 98: 1194–1199. 11158616 10.1073/pnas.031444798 PMC14731

[18] Medioni C, Bertrand N, Mesbah K, Hudry B, Dupays L, et al (2010) Expression of Slit and Robo genes in the developing mouse heart. Dev Dyn 239: 3303–3311. 20941780 10.1002/dvdy.22449 PMC2996720

[19] Mommersteeg MT, Andrews WD, Ypsilanti AR, Zelina P, Yeh ML, et al (2013) Slit-roundabout signaling regulates the development of the cardiac systemic venous return and pericardium. Circ Res 112: 465–475. 23255421 10.1161/CIRCRESAHA.112.277426

[20] Rothberg JM, Hartley DA, Walther Z, Artavanis-Tsakonas S (1988) slit: an EGF-homologous locus of D. melanogaster involved in the development of the embryonic central nervous system. Cell 55: 1047–1059. 3144436 10.1016/0092-8674(88)90249-8

[21] Spitzweck B, Brankatschk M, Dickson BJ (2010) Distinct protein domains and expression patterns confer divergent axon guidance functions for Drosophila Robo receptors. Cell 140: 409–420. 20144763 10.1016/j.cell.2010.01.002

[22] Evans TA, Bashaw GJ (2010) Functional diversity of Robo receptor immunoglobulin domains promotes distinct axon guidance decisions. Curr Biol 20: 567–572. 20206526 10.1016/j.cub.2010.02.021 PMC4078746

[23] Reuveny A, Elhanany H, Volk T (2009) Enhanced sensitivity of midline glial cells to apoptosis is achieved by HOW(L)-dependent repression of Diap1. Mech Dev 126: 30–41. 18984040 10.1016/j.mod.2008.10.004

[24] Yang L, Bashaw GJ (2006) Son of sevenless directly links the Robo receptor to rac activation to control axon repulsion at the midline. Neuron 52: 595–607. 17114045 10.1016/j.neuron.2006.09.039

[25] Hsouna A, Kim YS, VanBerkum MF (2003) Abelson tyrosine kinase is required to transduce midline repulsive cues. J Neurobiol 57: 15–30. 12973825 10.1002/neu.10232

[26] Hsouna A, VanBerkum MF (2008) Abelson tyrosine kinase and Calmodulin interact synergistically to transduce midline guidance cues in the Drosophila embryonic CNS. Int J Dev Neurosci 26: 345–354. 18243630 10.1016/j.ijdevneu.2007.12.005

[27] Ocorr K, Reeves NL, Wessells RJ, Fink M, Chen HS, et al (2007) KCNQ potassium channel mutations cause cardiac arrhythmias in Drosophila that mimic the effects of aging. Proc Natl Acad Sci U S A 104: 3943–3948. 17360457 10.1073/pnas.0609278104 PMC1820688

[28] Chanana B, Steigemann P, Jackle H, Vorbruggen G (2009) Reception of Slit requires only the chondroitin-sulphate-modified extracellular domain of Syndecan at the target cell surface. Proc Natl Acad Sci U S A 106: 11984–11988. 19574454 10.1073/pnas.0901148106 PMC2715486

[29] Knox J, Moyer K, Yacoub N, Soldaat C, Komosa M, et al (2011) Syndecan contributes to heart cell specification and lumen formation during Drosophila cardiogenesis. Dev Biol 356 2: 279–90. 21565181 10.1016/j.ydbio.2011.04.006

[30] Rodriguez-Fraticelli AE, Auzan M, Alonso MA, Bornens M, Martin-Belmonte F (2012) Cell confinement controls centrosome positioning and lumen initiation during epithelial morphogenesis. J Cell Biol 198: 1011–1023. 22965908 10.1083/jcb.201203075 PMC3444774

[31] Rhee J, Buchan T, Zukerberg L, Lilien J, Balsamo J (2007) Cables links Robo-bound Abl kinase to N-cadherin-bound beta-catenin to mediate Slit-induced modulation of adhesion and transcription. Nat Cell Biol 9: 883–892. 17618275 10.1038/ncb1614

[32] Hanai J, Gloy J, Karumanchi SA, Kale S, Tang J, et al (2002) Endostatin is a potential inhibitor of Wnt signaling. J Cell Biol 158: 529–539. 12147676 10.1083/jcb.200203064 PMC2173844

[33] Passos-Bueno MR, Suzuki OT, Armelin-Correa LM, Sertie AL, Errera FI, et al (2006) Mutations in collagen 18A1 and their relevance to the human phenotype. An Acad Bras Cienc 78: 123–131. 16532212 10.1590/s0001-37652006000100012

[34] Fukai N, Eklund L, Marneros AG, Oh SP, Keene DR, et al (2002) Lack of collagen XVIII/endostatin results in eye abnormalities. Embo J 21: 1535–1544. 11927538 10.1093/emboj/21.7.1535 PMC125362

[35] Wang X, Harris RE, Bayston LJ, Ashe HL (2008) Type IV collagens regulate BMP signalling in Drosophila. Nature 455: 72–77. 18701888 10.1038/nature07214

[36] Bunt S, Hooley C, Hu N, Scahill C, Weavers H, et al (2010) Hemocyte-secreted type IV collagen enhances BMP signaling to guide renal tubule morphogenesis in Drosophila. Dev Cell 19: 296–306. 20708591 10.1016/j.devcel.2010.07.019 PMC2941037

[37] Deng WM, Schneider M, Frock R, Castillejo-Lopez C, Gaman EA, et al (2003) Dystroglycan is required for polarizing the epithelial cells and the oocyte in Drosophila. Development 130: 173–184. 12441301 10.1242/dev.00199

[38] Harpaz N, Volk T (2011) A novel method for obtaining semi-thin cross sections of the Drosophila heart and their labeling with multiple antibodies. Methods 56: 63–68. 21963658 10.1016/j.ymeth.2011.09.017

[39] Gilsohn E, Volk T (2010) Slowdown promotes muscle integrity by modulating integrin-mediated adhesion at the myotendinous junction. Development 137: 785–794. 20110313 10.1242/dev.043703

[40] Ocorr K, Fink M, Cammarato A, Bernstein S, Bodmer R (2009) Semi-automated Optical Heartbeat Analysis of small hearts. J Vis Exp 10.3791/1435 PMC3150057 19759521

