# Supplementary figures and images for: Multiplexin Promotes Heart but Not Aorta Morphogenesis by Polarized Enhancement of Slit/Robo Activity at the Heart Lumen
Source: PLoS Genet. 2013 Jun 27;9(6):e1003597. doi: 10.1371/journal.pgen.1003597 (PMC3694841; doi:10.1371/journal.pgen.1003597)

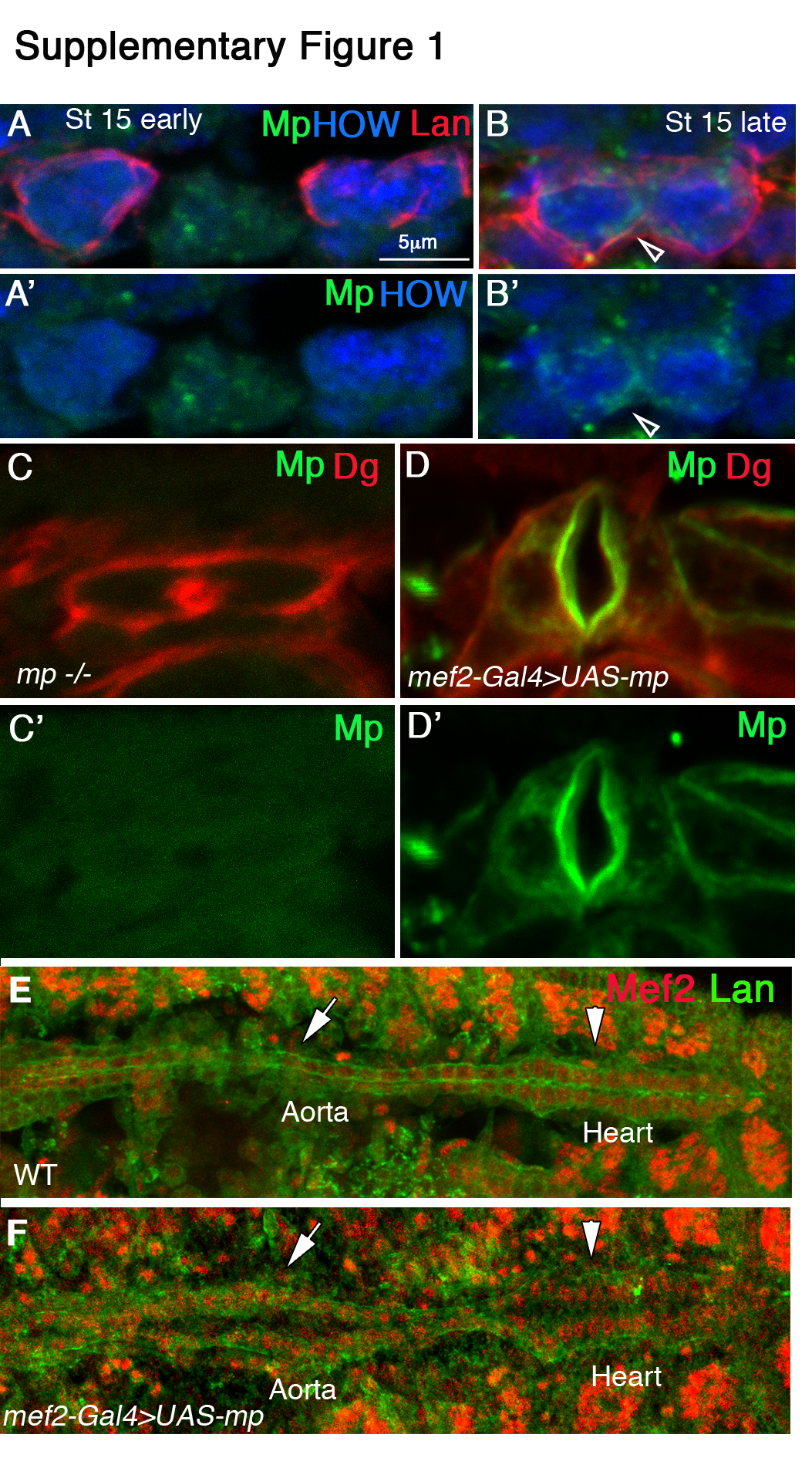

Supplement: Figure S1 — Mp distribution during heart tube formation, and its ability to expand the lumen of the aorta. Cross sections of cardioblasts at early stage 15 prior to cardioblasts encounter (A,A′) and following the formation of the dorsal junction (B,B′) stained for HOW (blue, marks the cardioblast cytoplasm), Mp (green) and Laminin (red). The arrowheads in B and B′ indicate the initial luminal secretion of Mp which partially overlaps Laminin staining. C,D Cross section of mp mutant heart (C,C′), or cardioblasts overexpressing Mp (D,D′), with anti Mp antibodies (green C–D′) and with anti Dg (red C,D). The specificity of the antibody is demonstrated by its negative staining in mp mutant. Note that overexpression of Mp in cardioblasts does not change its polarized luminal distribution (D). E,F Dorsal view of whole embryos labeled with anti Mef2 (red) and anti Laminin (lan, green). E-wild type embryo, F-embryo overexpressing Mp. Arrows indicate the aorta and arrowheads indicate the heart domains. Note the expansion of the aorta lumen following overexpression of Mp. (TIF) [file pgen.1003597.s001.tif]
